# Sharing the Space: Distribution, Habitat Segregation and Delimitation of a New Sympatric Area of Subterranean Rodents

**DOI:** 10.1371/journal.pone.0123220

**Published:** 2015-04-09

**Authors:** Bruno Busnello Kubiak, Daniel Galiano, Thales Renato Ochotorena de Freitas

**Affiliations:** 1 Programa de Pós-Graduação em Biologia Animal, Universidade Federal do Rio Grande do Sul, Porto Alegre, RS, Brazil; 2 Programa de Pós-Graduação em Ecologia, Universidade Regional do Alto Uruguai e das Missões—Campus de Erechim, Erechim, RS, Brazil; 3 Departamento de Genética, Universidade Federal do Rio Grande do Sul, Porto Alegre, RS, Brazil; University of Missouri Kansas City, UNITED STATES

## Abstract

Subterranean rodents of the genus *Ctenomys* usually present an allopatric or parapatric distribution. Currently, two cases of sympatry have been recognized for the genus in the coastal dunes of southern Argentina and southern Brazil. In this context, they are ideal models to test hypotheses about the factors that delimit the patterns of space use and to understand interspecific interactions in small mammals. We investigated the vegetation structure, plant biomass and soil hardness selected by two species of subterranean rodents (*Ctenomys flamarioni* and *C*. *minutus*) when distributed in sympatry and allopatry from nine different areas along the line of coastal dunes in southern Brazil. In addition, our work presents a new record of a third area of sympatry for the genus *Ctenomys*. *Ctenomys flamarioni* and *C*. *minutus* show habitat segregation in the area where they occur in sympatry. These species show segregation in their selection of microhabitats, differing in relation to soil hardness, plant biomass, and plant cover. *Ctenomys flamarioni* showed a distinction in habitat selection when occurring in allopatry and sympatry, whereas *C*. *minutus* selected the same habitat characteristics under both conditions. A possible explanation to the observed pattern is that these species have acquired different adaptations over time which allows them the ability to exploit different resources and thus avoid competitive interactions all together.

## Introduction

The organization and spatial distribution of organisms play important roles in population stability, thereby directly influencing species coexistence [1]. Biological factors, such as interspecific interactions, may play a key role in the distribution of subterranean rodents [2] and may substantially influence community structure [3]. Different species of subterranean rodents are rarely sympatric, possibly because their morphological similarities make resource partitioning and subdivision of the subterranean niche difficult [4–5]. Instead, these species tend to have allopatric distributions, leading to the initial conclusion that congeneric competition has a minor effect on subterranean rodent populations [6–7]. Subterranean rodents of the genus *Ctenomys* (tuco-tucos) are characterized by a limited individual mobility and a patchy distribution of the local populations [5]. Due to these characteristics, they are ideal models to test hypotheses about the factors that delimit the patterns of space use and to understand interspecific interactions in small mammals [8].

Species from the genus *Ctenomys* are widely distributed throughout South America [5, 9]. Until now, two cases of sympatry have been recognized for the genus. The first is widely recognized and studied and occurs between the species C. *australis* Rusconi, 1934 and *C*. *talarum* Thomas, 1989 [9–10] in a coastal dune region in the southern Buenos Aires province of Argentina. These species show segregation in their selection of microhabitats, differing in relation to soil and vegetation characteristics [2, 8, 11]. The second case of sympatry was reported to occur in the southern Brazil coastal plain between *C*. *flamarioni* Travi, 1981 and *C*. *minutus* Nehring, 1887 [12]. However, this sympatric zone has been neglected and there is no information regarding the extent of the area, the environmental characteristics or the habitat segregation among species in this location. *C*. *flamarioni* is narrowly distributed in the first range of mobile sand dunes of this coastal plain of southern Brazil [12]. *Ctenomys minutus* inhabits the sandy fields and dunes of the region and occupies the largest latitudinal gradient among the tuco-tucos in southern Brazil [12–13]. To the south of its distribution, *C*. *minutus* is found strictly on sandy fields, approximately 2 km from the coast. This species is also found on the range of coastal dunes near Capão da Canoa (29°46’00”S, 50°00’58” W), where it comes into contact with *C*. *flamarioni* [12].

Information on interspecific interactions among ctenomyids in sympatric areas has been documented in other studies [2, 8, 11]. However, these studies only provided information about habitat segregation in the sympatric areas and did not evaluate how species relate to habitat characteristics when distributed in allopatry. In general, these studies demonstrated that species segregate the environment according to the soil hardness, plant cover and plant biomass. We propose that these relationships of habitat segregation could be better understood by evaluating the environmental characteristics of both species when distributed in allopatry and by comparing this pattern with individuals with sympatric distribution. For this purpose, we evaluated different areas within the distribution of *C*. *flamarioni* and *C*. *minutus* by considering areas where the species occur in sympatry and areas where the species occur in allopatry. In addition, this work presents new information about the limits of the sympatric area cited by Freitas [12] and identifies a third sympatric area for the *Ctenomys* genus.

## Materials and Methods

### Animal capture and delimitation of the sympatric area

**Fig 1:**
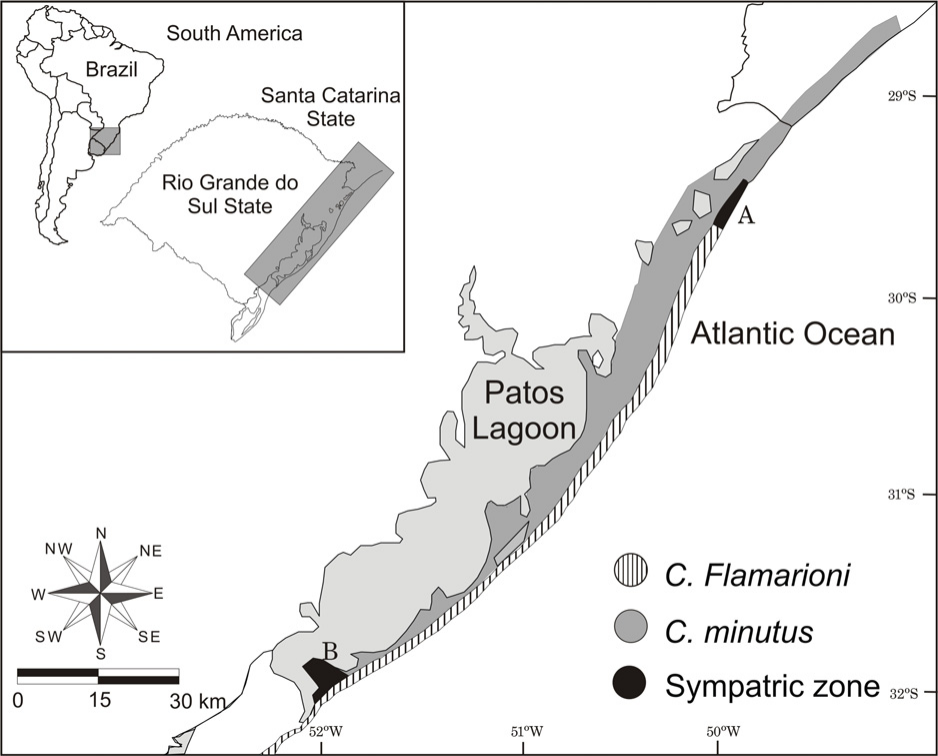
Geographic distribution of *C*. *flamarioni* and *C*. *minutus* in the coastal plain of southern Brazil. Two sympatric areas are indicated in the map by letters: A—sympatric area cited by Freitas (1995); B—new sympatric area described by these species. South America map obtained from OpenStreetMap (free available at: http://www.openstreetmap.org/). The image was edited using CorelDraw graphics Suite.

Specimens of *C*. *flamarioni* and *C*. *minutus* were sampled from nine different areas along the line of coastal dunes in southern Brazil: three areas where the species occur in sympatry (29°43‘05S, 49°59‘22W; 29°42‘20S, 49°58‘58W; 29°41‘30S, 49°58‘32W), three areas where *C*. *flamarioni* presents allopatric distribution (32°43‘54S, 52°27‘18W; 29°48‘56S, 50°02‘31W; 29°47‘39S, 50°01‘44W), and three areas where *C*. *minutus* presents allopatric distribution (29°34‘12S, 49°53‘54W; 29°29‘21S, 49°50‘20W; 29°23‘44S, 49°46‘02W). Each area was defined according to the total width of the dunes and had a maximum length of 200 meters. The sampling areas were at least one kilometer apart from each other (Fig 1).

The sampling was performed in two different campaigns between the months of January and February 2012 and between October and November 2012. In each area, we sampled a total of 10 individuals, and in the area of sympatry, five individuals of each species were collected. A total of 45 individuals of *C*. *flamarioni* (30 distributed in allopatry and 15 sympatrically) and 45 *C*. *minutus* (30 distributed in allopatry and 15 sympatrically) were sampled. The animals were captured using Oneida Victor No. 0 traps protected with rubber strips to capture the animals without injury. Each trap was introduced into the entrance of a burrow and checked every 10 minutes to reduce stress to the animals caught. This procedure was conducted only to confirm the presence of an animal in the burrow, and all individuals were then released at their respective trapping locations less than five minutes of the capture event.

Additionally, we determined the geographical limits of the sympatric area by sampling the tuco-tucos in a buffer region of 40 km between the south-north known boundaries proposed by Freitas [12] (south: 29°58’20.01” S, 50°07’00.94”W; north: 29°38’46.59”S, 49°56’52.2”W). This procedure allowed us to determine the exact region where both species get in contact. Animals were captured with the same methodology as above. Each captured animal was released at their point of capture and all of the coordinates were recorded using a GPS (Garmins-Vita). This study was carried out in strict accordance with the recommendations of the American Society of Mammalogists [14]. The protocol was approved by the IBAMA—Brazilian Institute for the Environment and for Renewable Natural Resources (Permit number: 14690–1). This work was not submitted to an institutional Animal Ethics Committee because none of the animals would be injured or sacrificed.

### Habitat characteristics and statistical analysis

To evaluate the vegetation and soil characteristics, we considered every point where an individual was captured as a central point, following the methodology proposed by Galiano et al. [15]. At each central point, vegetation structure, plant biomass, and soil hardness were sampled in four sampling units (SU) situated at a distance of 2 m in the four cardinal directions. To evaluate vegetation structure, we used a 1-m^2^ quadrat to measure plant cover of grasses and herbs. Plant biomass was measured in these same quadrats with a vertical quadrat sampler with a side length of 16 cm and a side depth of 22 cm (one sample per SU). We removed all above- and below-ground vegetation from inside this quadrat sampler. After removing sand, plant samples were oven-dried to a constant weight at 80°C, and then dry weight was recorded to determine total biomass. We used an impact soil penetrometer (Model IAA / Planalsucar, [16]) to measure soil hardness. At each sampling point, we took four measurements (one per SU), recording the number of strokes necessary to penetrate into the first 50 cm of soil (results are expressed in kg/cm^2^). For analysis of vegetation variables and soil hardness, we computed the mean of the four sampling units and then used the mean in the analysis. For plant biomass, the weights of the four samples per unit were summed for the subsequent analysis.

Considering that not all variables had normal distribution, we used a permutational ANOVA analysis (PERMANOVA) to compare habitat characteristics (plant biomass, plant cover, and soil hardness) between species. Each species and condition (allopatry or sympatry) was considered a block in all analyses. The same analysis was used to check for differences in habitat characteristics when species were distributed in sympatry and in allopatry. The tests were performed with matrices based on Euclidean distances, and we ran 9999 permutations. The associations of habitat characteristics used by each species in the sympatric area were assessed using a principal component analysis (PCA) based on a covariance matrix that used the habitat variables collected from the trapped animals. In the results, we present only the information and loadings of the first axis (PC1), since PC2 and other axes had less relevant explanations (≤7%). A Wilcoxon rank test was used to compare the scores of *C*. *flamarioni* and *C*. *minutus* for the PC1. All analyses were conducted with the vegan package [17] in the R program for statistical computing [18].

## Results

### Delimitation of the sympatric area

The sympatric area where *C*. *flamarioni* and *C*. *minutus* are distributed is located in the first range of dunes on the coastal plain of Rio Grande do Sul state in southern Brazil, and covers a length of approximately 15 km. Both species only come into contact in the first range of dunes that extend from Capão da Canoa in the south (29°46’04.41”S, 50°00’59.1”W) to Arroio Teixeira in the north (29°38’23.83”S, 49°56’36.77”W). This area is characterized by the habitat transition of *C*. *minutus*. In the southern portion of this region, this species is only found in sandy fields, mostly in the interior of the continent, whereas in the northern portion, this species also occupies the dune environment. Moreover, another sympatric area between these species was observed in the Coastal Plain of the Rio Grande do Sul state, but it is located in the southern portion towards the coast. Only a few records of individuals were obtained in the area, in the São José do Norte municipality (32°03'18.15”S, 51°59'43.94”W). This sympatric zone does not occur in the dunes, as both species come into contact in a region of sandy fields. In this area, *C*. *flamarioni* occupies the environment where previously there were only records of *C*. *minutus* (from the sandy fields up to the shores of the Patos Lagoon) (Fig 1).

### Habitat characteristics and spatial distribution of the species

**Fig 2:**
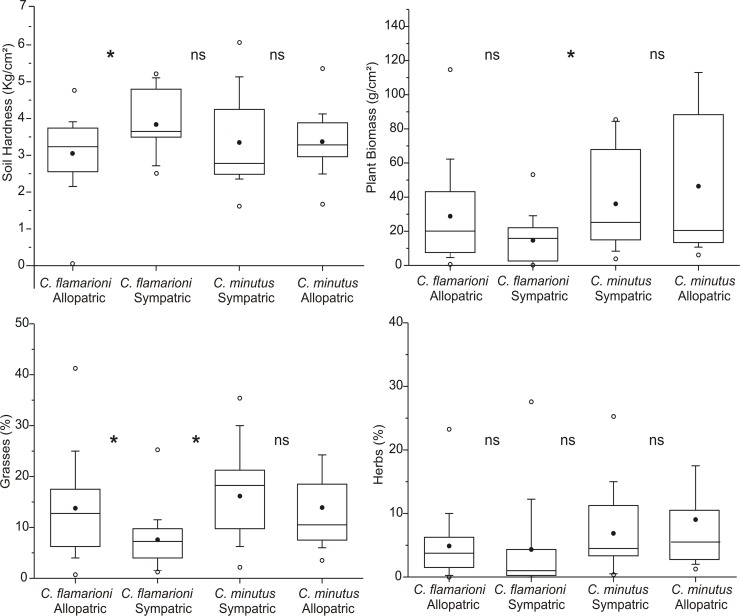
Comparisons of soil hardness, plant biomass, grass cover and herb cover between *C*. *flamarioni* and *C*. *minutus* distributed in sympatric and allopatric areas. Top and lower horizontal lines of boxes represent standard deviations. Black points and the center horizontal lines represent the mean and the median, respectively. The results of the statistical tests are displayed in the text. Asterisk indicates a significant difference and “ns”non-significant. For all the variables, the middle “*/ns” reflects a test between species, and the first and third “*/ns” reflect comparisons within species.

*Ctenomys flamarioni* and *C*. *minutus* use different habitat characteristics when they occur in sympatry. *Ctenomys minutus* selects areas with higher amounts of plant biomass and grass cover relative to the areas occupied by *C*. *flamarioni* (*F* = 6.45, *p* = 0.015 and *F* = 9.32, *p* = 0.005, respectively). However, there is no difference in the selection of areas in relation to soil hardness and herb cover (*F* = 1.58, *p* = 0.22 and *F* = 0.96, *p* = 0.34, respectively) (Fig 2).

With respect to the differentiation in the selection of habitats, we observed that when *C*. *flamiarioni* occurs in allopatry, this species selects areas with high grass cover and are distributed on less hard soils in comparison to individuals of the same species located in the sympatric zone (*F* = 5.819, *p* = 0.018 and *F* = 7.507, *p* = 0.008, respectively). On the other hand, there is no difference in the environmental characteristics of the habitats selected by *C*. *minutus* individuals when they occur in sympatry or in allopatry (Fig 2).

**Fig 3:**
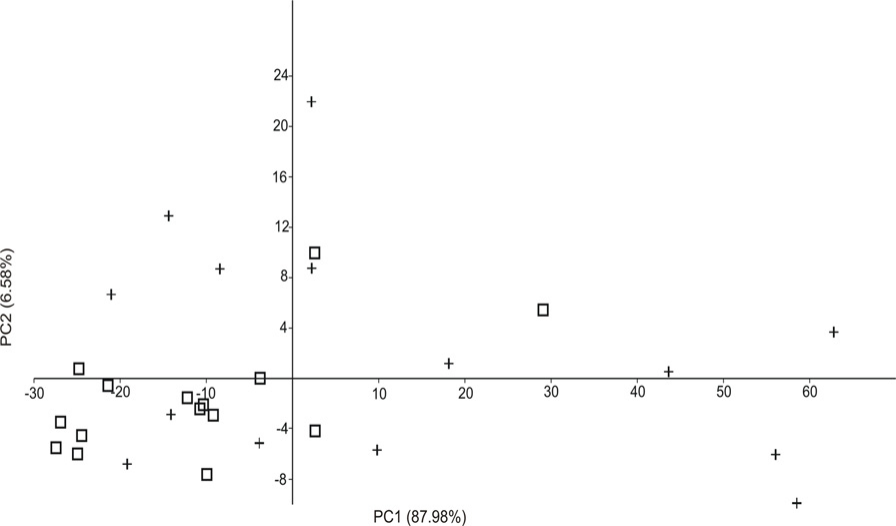
Principal component analysis (PCA) of habitat features selected by *C*. *flamarioni* (□) and *C*. *minutus* (+) species in sympatric area in the southern Brazilian coastal plain.

The PCA results showed that plant biomass and grass and herb cover were positively correlated with the PC1 axis (factor loadings: 0.9717, 0.2151 and 0.095, respectively). Plant biomass had the highest correlation value with this axis, in contrast to soil hardness, which showed a negative correlation (factor loading: -0.0190). The PC1 axis explained 87.98% of the total variation. Through the PCA results, it was possible to identify two groups; the first group consisted of *C*. *minutus* individuals, characterized by being distributed in places with higher plant cover and biomass, and the second group, formed by *C*. *flamarioni*, was found in places with lower plant cover and biomass (Fig 3). We found a significant difference between the scores of *C*. *flamarioni* and *C*. *minutus* for PC1 axis (*Z* = 1.931, *p* = 0.0267).

## Discussion

*Ctenomys minutus* and *C*. *flamarioni* show habitat segregation in the area where they occur in sympatry. This has already been shown in congeneric species, such as *C*. *australis* and *C*. *talarum* [2, 8, 11], and other species of subterranean rodents [19–21]. The habitat segregation described for species of subterranean rodents is most likely related to the similarity of the species’ habits [5], as closely related species often devise strategies to avoid competition [22–23]. In the present study, we observed that individuals of *C*. *flamarioni* select different sites in the sympatric zone from the places commonly used when they are distributed in allopatry. However, *C*. *minutus* does not show any changes in the selected environments when it occurs in sympatry or allopatry. Several studies demonstrate interspecific competition as one of the main causes of allopatric distribution (usually found in subterranean rodents), where the species that presents competitive superiority selects the habitat of its preference, suppressing other species which occupy less favorable habitats [19, 20, 24, 25]. Our results suggest that *C*. *minutus* selects environments with the same characteristics (soil hardness, biomass, and plant cover) in both sympatric and allopatric distributions. Thus, it seems that *C*. *flamarioni* individuals might be pushed into different environments than they usually use when found in allopatry.

Moreover, Malizia et al. [11] demonstrated that in the sympatric area of tuco-tucos in Argentina, *C*. *australis*, originally distributed in the coastal dune region, is the dominant species in the dune environment. Vassallo [26] observed that interspecific interactions and displacement by *C*. *australis* could be significant but nonetheless marginal in sustaining the pattern of microhabitat segregation between the species. These differences may be related to physiological factors, where the largest species present a better thermoregulatory capacity and a lower resting metabolic rate, as reported for other subterranean rodents [27, 28]. In our case, the species which has a mean larger body size is *C*. *flamarioni* in relation to *C*. *minutus* (266 mm ± 30.98 and 250 mm ± 35.13, respectively; t = 3.45, *p* = 0.0004; information based on records from the collection of the Laboratório de Citogenética e Evolução of the Universidade Federal do Rio Grande do Sul). However, we did not find the same pattern described for *C*. *australis* and *C*. *talarum*. Thus, it is possible to assume that other factors, such as aggressiveness, might be responsible for the observed pattern. The aggressive behavior in subterranean rodents is a known mechanism that contributes to competitive exclusion [3]. This behavior was studied among ctenomyids in laboratory (*C*. *australis* and *C*. *talarum*), with the larger-bodied *C*. *australis* dominating the smaller-bodied (see [29] for more details). Similarly, Hickman [30] recorded that *Thomomys bottae*, *Geomys bursarius*, and *Pappogeomys castanops* present aggressive interactions, with larger individuals being behaviorally dominant in interspecific encounters.

Nevertheless, the distribution of these two species in the coastal plain may be related to historical factors of occupation of the range of coastal dunes that they inhabit. During the development of the coast of southern Brazil in the Pleistocene and early Holocene, four barrier systems arose, related to four transgressive and regressive events [31, 32]. The first and second barriers appeared at the beginning and middle of the Pleistocene, respectively. Then, at late Pleistocene, a third barrier increased the Coastal Plain, forming a multiple barrier system, which is inhabited by the species *C*. *minutus* [31, 32, 12]. Finally, at the beginning of the Holocene, the last movement originated the fourth barrier, corresponding to the first dune line, and extends along the entire coastline of the states of Rio Grande do Sul and southern Santa Catarina [31,32], which is occupied by *C*. *minutus* above the sympatric zone, and by *C*. *flamarioni* below this contact region. Moreover, the evolutionary history of the species shows that *C*. *flamarioni* is closely related to the species present in Uruguay and Argentina, and show a pattern of occupation South-North [12, 33,34], whereas *C*. *minutus* shows a pattern of North-South occupation, from Santa Catarina to Rio Grande do Sul state [35]. Consequently, the geological development of the coast of southern Brazil associated with the evolutionary history of both species may have had a significant influence in the way that these species are distributed nowadays.

Our results demonstrate that the vegetation characteristics (plant biomass and grass cover) appear to be closely related to the habitat segregation of *C*. *flamarioni* and *C*. *minutus*. Between the characteristics of the vegetation, grass cover appears to be the most important variable for both species, probably related to the fact that grasses are the major food source for both species [15, 36, 37]. Additionally, Lopes et al. [36] demonstrated that thirteen plant families, mainly represented by grasses, were identified in the diet of *C*. *minutus*, and 10 families, also with a predominance of grasses, were present in diet of *C*. *flamarioni*. These authors found differences in diet composition between species, suggesting some level of diet partitioning between *C*. *flamarioni* and *C*. *minutus* in the sympatric region. They concluded that these differences might have been developed to avoid competition in the region of co-occurrence (see Lopes et al. [36] for more details). Additionally, vegetation not only supplies food but also affects patterns of ventilation and heat flow within burrow systems [38]. Therefore, the environmental characteristics related to vegetation may have a significant influence on the spatial distribution of subterranean rodents when distributed in sympatry.

Although our study species occur in sympatry, they are segregated microspatially by different habitat preferences, and the observed vegetation and soil preferences might be related to the quality and quantity of food in the inhabited area. Because the tuco-tucos occur in a coastal environment, the relationship between soil hardness and plant cover contrasts with other variables not measured in this study. One possible explanation to the observed pattern is that these species have acquired different adaptations over time which allows them the ability to exploit different resources [39] and thus avoid competitive interactions all together. It should be emphasized that the sympatric area located in the south was not included in our analysis considering that there are no records of allopatric distribution of *C*. *flamarioni* in sand fields. Nevertheless, a great sampling effort is still needed to characterize the limits of this second area and to verify the relationship between the spatial distribution of this species and its habitat characteristics.
